# Landscape response to progressive tectonic and climatic forcing in NW Borneo: Implications for geological and geomorphic controls on flood hazard

**DOI:** 10.1038/s41598-017-00620-y

**Published:** 2017-03-28

**Authors:** David Menier, Manoj Mathew, Manuel Pubellier, François Sapin, Bernard Delcaillau, Numair Siddiqui, Mu. Ramkumar, M. Santosh

**Affiliations:** 1 Université Bretagne Sud, Laboratoire Géosciences Océan, UMR CNRS 6538, rue Yves, Mainguy, 56017 Vannes cedex France; 2 Ecole Normale Supérieure UMR 8538 du CNRS, 24 rue Lhomond, 75231 Paris, Cedex 05 France; 3 Total E&P MENA Dubai, The H-Dubai Office Tower, 24th Floor, 1, Sheikh Zayed Road, P.O. Box, 116538 Dubai, United Arab Emirates; 4 Université de Caen. UMR 6143 M2C, Morphodynamique continentale et côtière 24, Rue des tilleuls, 14000 Caen cedex France; 5 Universiti Teknologi PETRONAS Faculty of Geosciences & Petroleum Engineering, Bandar Seri Iskandar, 31750 Tronoh, Perak, Malaysia; 6 Periyar University Department of Geology, 636011 Salem, India; 7 University of Adelaide, Department of Earth Sciences, Adelaide, SA 5005 Australia; 8 China University of Geosciences, Beijing School of Earth Sciences and Resources 29 Xueyuan Road, Beijing, 100083 China

## Abstract

Empirical models have simulated the consequences of uplift and orographic-precipitation on the evolution of orogens whereas the effects of these forcings on ridgelines and consequent topography of natural landscapes remain equivocal. Here we demonstrate the feedback of a terrestrial landscape in NW Borneo subject to uplift and precipitation gradient owing to orographic effect, and leading to less-predictable flooding and irreversible damages to life and property. Disequilibrium in a large catchment recording the lowest rainfall rates in Borneo, and adjacent drainage basins as determined through χ, a proxy for steady–state channel elevation, is shown to result in dynamic migration of water divide from the windward-side of the orogen towards the leeward-side to attain equilibrium. Loss of drainage area in the leeward-side reduces erosion rates with progressive shortening resulting in an unstable landscape with tectonic uplift, gravity faults and debris flows. ^14^C dating of exhumed cut-and-fill terraces reveal a Mid–Pleistocene age, suggesting tectonic events in the trend of exhumation rates (>7 mm a^−1^) estimated by thermochronology, and confirmed by morphotectonic and sedimentological analyses. Our study suggests that divide migration leads to lowered erosion rates, channel narrowing, and sediment accretion in intermontane basins on the leeward-side ultimately resulting in enhanced flooding.

## Introduction

**Figure 1:**
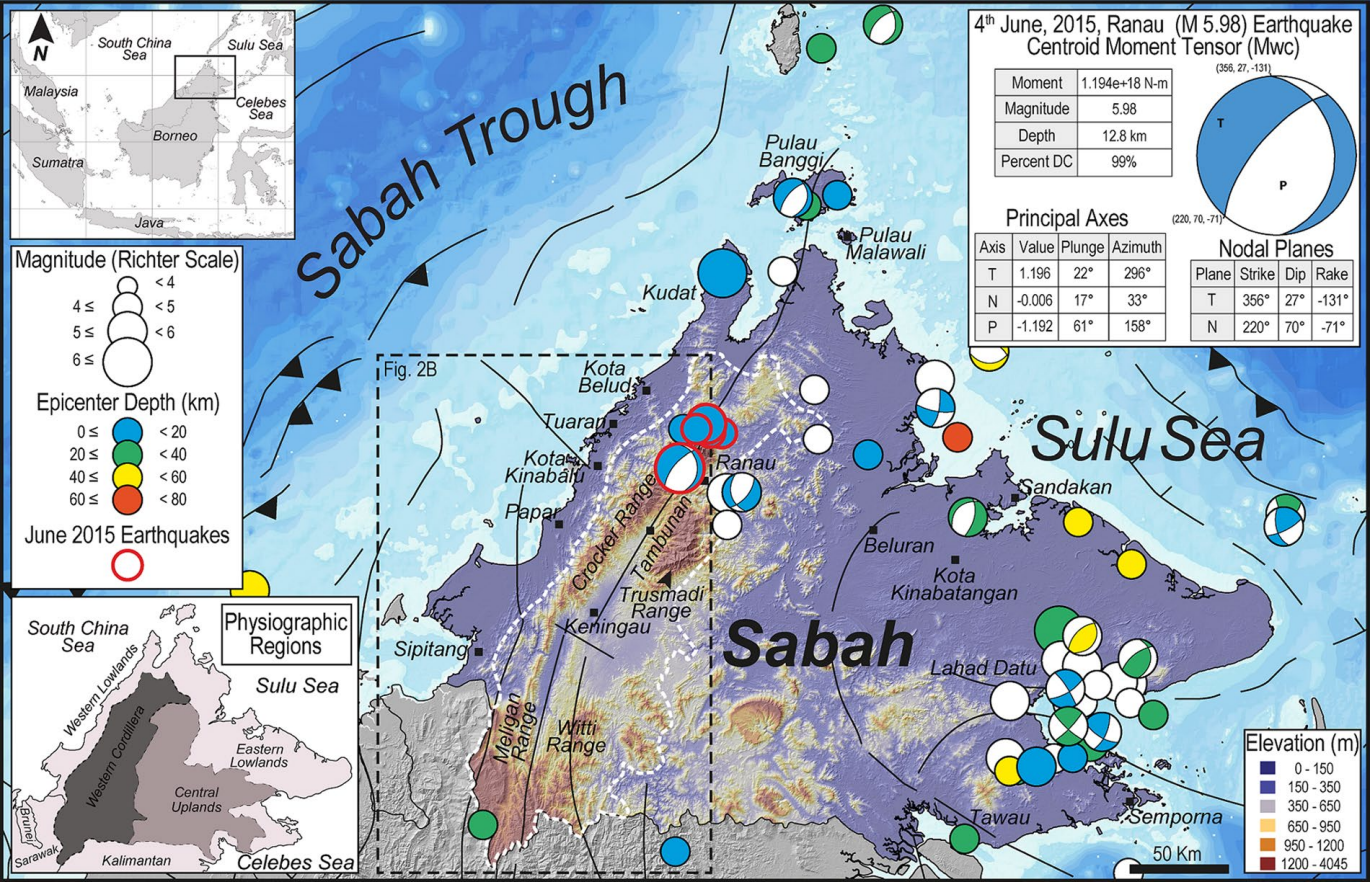
Location map of the study area in Sabah, NW Borneo. Shaded relief model of Sabah and main earthquake locations from 1911 to 2009 ^102^ are depicted by circles of different sizes highlighting magnitude on the Richter scale. Variation in fill color of the circles denotes depth of the epicenter. Earthquakes of June 2015 that originated from a depth of 12 km are indicated by circles with red outline. White dashed-line delineates the Western Cordillera. Major faults are shown in black and are based on Pubellier and Morley^103^. Base map is a SRTM^104^ (Shuttle Radar Topographic Mission) Digital Elevation Model (DEM) of 30 m (1-arc second) spatial resolution (SRTM 1 Arc-Second Global elevation data courtesy of the U.S. Geological Survey, https://lta.cr.usgs.gov/SRTM1Arc). The main map and inset maps were created using Geographic Information Systems (GIS) software ESRI ArcGIS^105^ version 10.2.1 (http://www.esri.com/software/arcgis/arcgis-for-desktop). Figure labels were added using Adobe Illustrator^106^ version CS5.1 (http://www.adobe.com/products/illustrator.html).

The continued, yet variable rates of interactions between tectonic and climatic forcing result in diversity of landscape evolution. A number of physical and numerical models^1–4^ have shown the effects of uplift and orographically enhanced precipitation on the evolution of active mountain ranges. However, in a natural landscape, subject to tectonic uplift and climatic perturbations, the feedback of ridgelines and subsequent rain-shadowed topography remains less-understood. These natural processes, often aggravated by anthropogenic intervention can result in catastrophic geohazards such as flooding, causing irreversible damages. The consequences of floods include loss of human life, crops and livestock, and the spread of water borne diseases^5–7^. Economic vulnerability arising from damage to infrastructure such as roads and bridges could have long–term impacts causing disruption to transportation and emergency flood evacuation services. Thus, understanding the consequences of varied landscape evolution under the influences of tectonic and climatic forcing has critical inputs for mitigation of geohazards especially in tectonically dynamic, climatically sensitive and highly populated regions of the World such as Southeast Asia. NW Borneo (Fig. 1) is a typical example where floods have caused community disruption and economic loss, increased health risks, and has claimed numerous lives^8, 9^. With the rapid industrialization and urbanization in the entire Sabah, the insufficiency of land has led to enhanced exposure of lives and infrastructure to potential flood hazard.

Northwest Borneo, located on the edge of the Sunda Plate, is seismically active as illustrated by the recent occurrence of magnitude 6.0 earthquake in the Sabah region on June 5, 2015. The average magnitude of earthquakes in the region is above four on the seismic scale (USGS database) (Fig. 1), although spatial geodesy does not show any substantial motion between central Borneo and the Sunda Plate^10^. The tectonic evolution of the NW Borneo Trough involved the subduction of the oceanic crust of the proto-South China Sea beneath Sundaland during the Middle Eocene. Subduction ceased during the Early Miocene^11, 12^ and was followed by collision^13^, when the thinned continental crust of the Dangerous Grounds began to subduct beneath the Borneo mainland. The NE-SW oriented tectonic belt of NW Borneo is parallel to the nearby Sabah Trough and is related to the ‘Sabah Orogeny’ with subduction and collision of the Dangerous Ground Continental Block with NW Borneo^13–16^ (Fig. 1). NW Borneo was tectonically active until the Middle Miocene and Borneo is recognized to have experienced an intraplate setting since Late Miocene. The region underwent constant and continuous uplift owing to the break-off of the subducted slab below Sabah, NW Borneo^17^. Other explanations for the cause of recent tectonics onshore and offshore NW Borneo include subduction in the NW Borneo Trough until the Late Neogene or present-day^18, 19^, regional compression^16, 20, 21^, or extension^22^, ongoing convergence of blocks or plates, inheritance from former subduction or far-field stresses^17, 23, 24^, and possible large-scale mantle processes. Reactivation of the frontal thrust has also been recently reported^10^.

**Figure 2:**
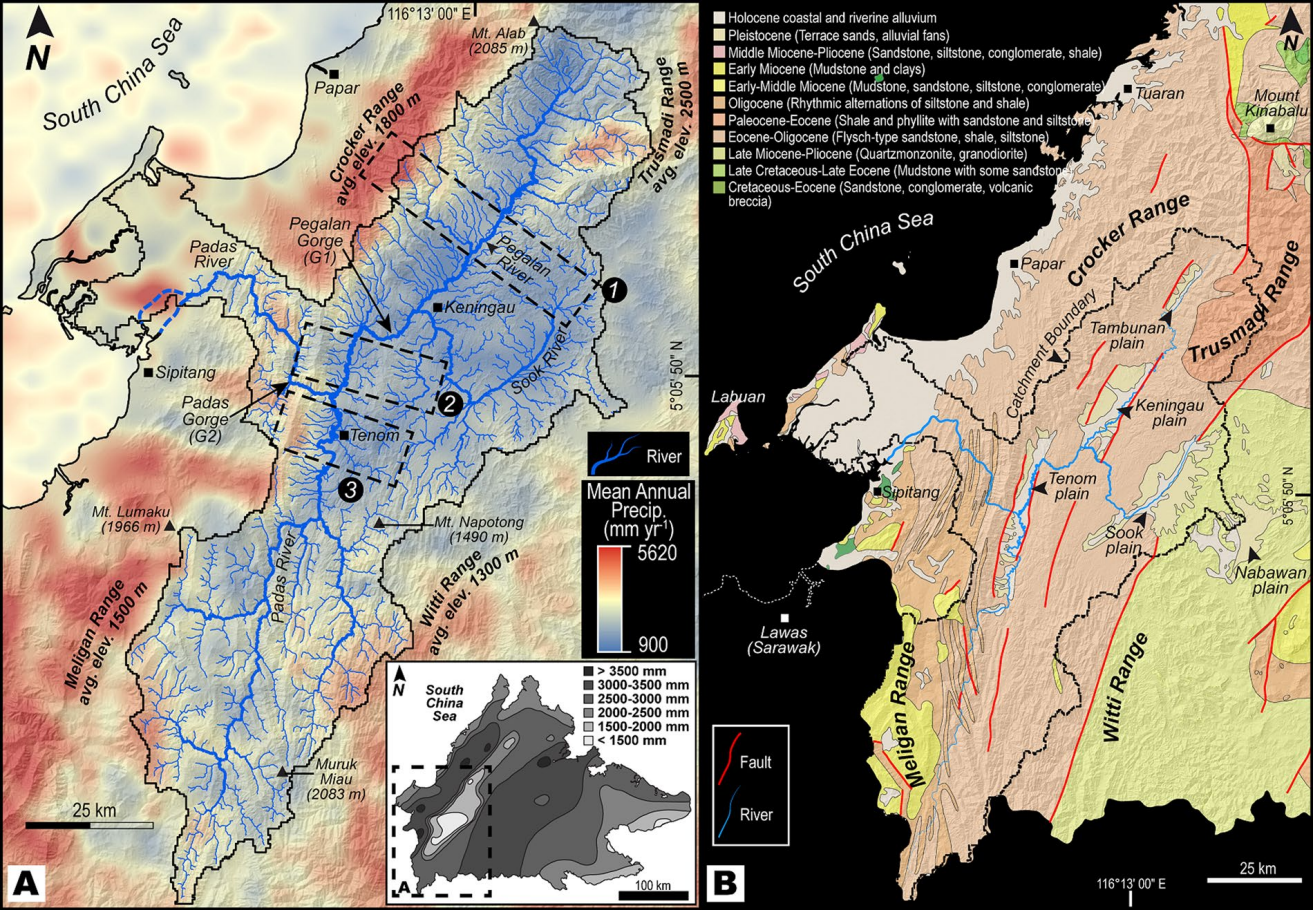
Geography and geology of the study area. (**A**) Mean annual precipitation for the studied catchment and surrounding orogenic belts derived from TRMM^95^ (Tropical Rainfall Measurement Mission) data. Note the orographically enhanced precipitation over the four mountain ranges and the sheltered rain-shadowed catchment with an area of 8800 km^2^ demonstrating dry conditions. The catchment incorporates four plains (Tambunan, Keningau, Sook and Tenom Plains) filled with Quaternary sediments. Tambunan, Keningau and Sook Plains confluence with the Padas river in the Tenom Plain via a narrow gorge G1 (Pegalan Gorge). The Padas River ultimately drains into the South China Sea via the Padas Gorge (G2). Dashed-lines numbered 1–3 depict lateral limit of rectangular swaths of 10 km width utilized for compressing topographic information into a single profile. Inset map shows spatial distribution of precipitation of entire Sabah, NW Borneo. (**B**) Simplified geological map of the studied drainage basin and surrounding areas along with major faults and structural lines. Apart from minor amounts of volcanic rocks of the Mount Kinabalu, the dominant lithologies are relatively lesser resistant sedimentary rocks. Faults are based on Pubellier and Morley^103^. Base map in (**A**) and (**B**) is a SRTM^104^ (Shuttle Radar Topographic Mission) Digital Elevation Model (DEM) of 30 m (1-arc second) spatial resolution (SRTM 1 Arc-Second Global elevation data courtesy of the U.S. Geological Survey, https://lta.cr.usgs.gov/SRTM1Arc). The maps were created using Geographic Information Systems (GIS) software ESRI ArcGIS^105^ version 10.2.1 (http://www.esri.com/software/arcgis/arcgis-for-desktop). Figure labels in (**A**) and (**B**) were added using Adobe Illustrator^106^ version CS5.1 (http://www.adobe.com/products/illustrator.html).

**Figure 3:**
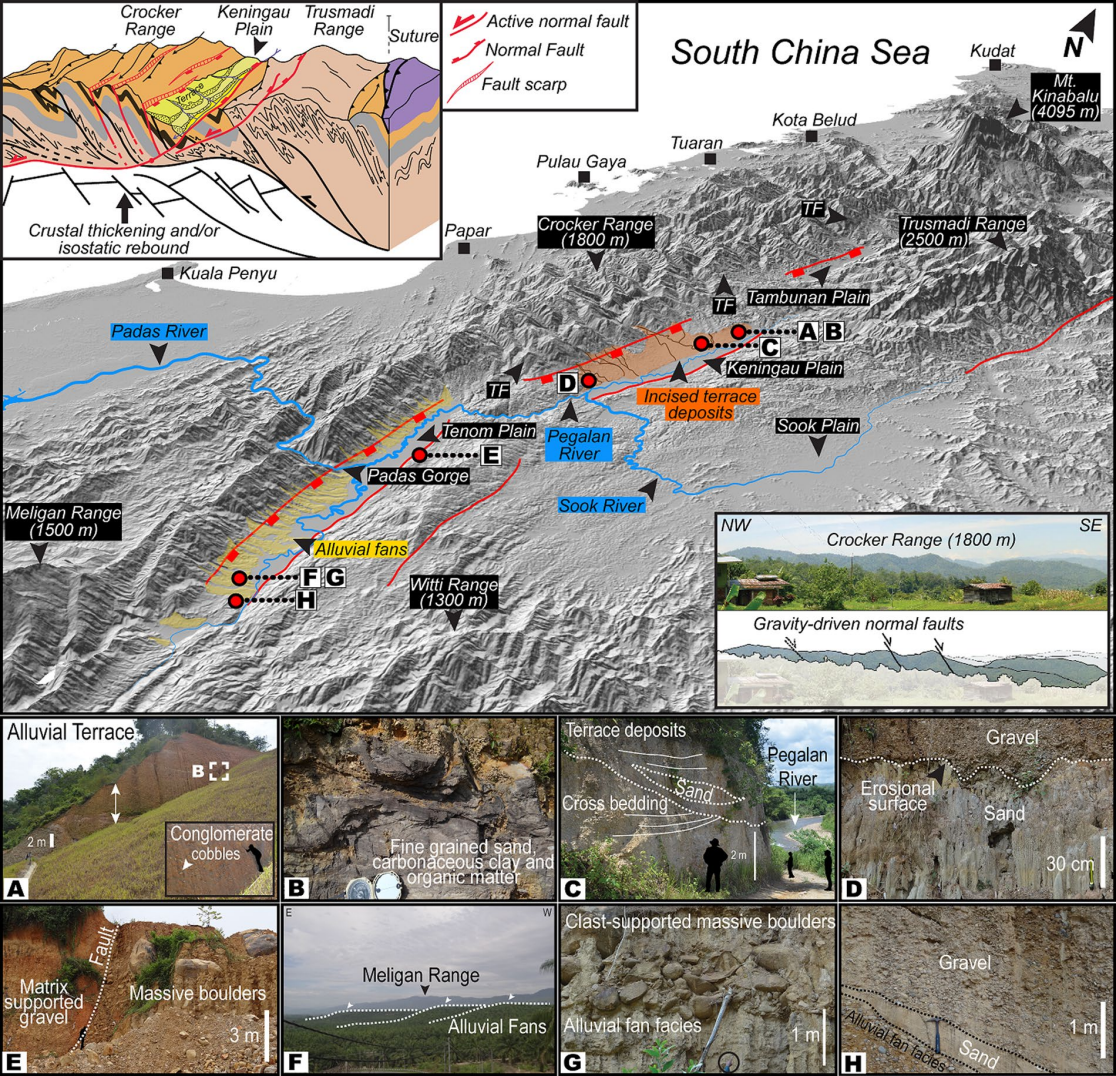
Perspective view of the landscape of NW Borneo. Salient topographic and geomorphic features of the study location. Note alluvial fans in the Tenom Plain and incised terrace deposits in the Keningau Plain located along strike of the known faults. Also shown are locations of fieldwork and corresponding photographs (**A**–**H**) highlighting some identified sedimentary structures and lithofacies. Refer to text and Table 2 for details. Photograph B indicates location of sampling of organic material (wood) collected for radiocarbon dating. Perspective view is constructed from a SRTM^104^ (Shuttle Radar Topographic Mission) Digital Elevation Model (DEM) of 30 m (1-arc second) spatial resolution (SRTM 1 Arc-Second Global elevation data courtesy of the U.S. Geological Survey, https://lta.cr.usgs.gov/SRTM1Arc). The map was created using Geographic Information Systems (GIS) software ESRI ArcGIS^105^ version 10.2.1 (http://www.esri.com/software/arcgis/arcgis-for-desktop). Figure labels were added using Adobe Illustrator^106^ version CS5.1 (http://www.adobe.com/products/illustrator.html).

The climate of NW Borneo is equatorial with relatively uniform temperatures in the range of 27 to 32 °C, high humidity (80–85%), and copious rainfall (>3000 mm yr^−1^) (Fig. 2A). The northeast monsoon from November to March and the southwest monsoon between May and September exert a dominant influence on the climate of this region. In contrast to the copious rainfall received throughout NW Borneo, the structural architecture shelters an elongated intermontane basin owing to orographic effect with relatively low rates of precipitation (900–1400 mm yr^−1^) within the basin (Fig. 2A). The basin is bounded by several mountain ranges (Crocker, Trusmadi, Meligan and Witti Ranges) (Fig. 2A and B) that have been dramatically uplifted and eroded mainly in the Late Miocene^13, 17, 25–27^. The intermontane basin hosts four Quaternary plains predominantly underlain by Paleogene and Neogene sedimentary rocks that were deposited in the paleo-NW Borneo Trough and displays varying geomorphic characteristics and features^13^(Figs 2B and 3).

In NW Borneo, flood-prone areas are under heavy development albeit major flash floods and/or basin-wide floods in recent years^28^. Regardless of the low amount of annual precipitation in the intermontane basin under study, this area has been declared as a flood hazard zone^28^ with a single outlet for the rivers draining the four Quaternary plains covering an area of 8800 km^2^ (Figs 2 and 3). Although flooding has been globally attributed to excessive amounts of rainfall among other lesser contributing factors (e.g., ground cover, tidal influences, flat topography of coastal zones, ruptured dams etc.), the geologic and geomorphic evolution of a landscape as a principal contributing element to flooding in areas receiving low annual precipitation has received less attention.

Here we report the feedback of a terrestrial landscape in NW Borneo subject to tectonic uplift and lateral precipitation gradient resulting from orographic effect. We utilize a quantitative and qualitative approach in order to elucidate the controls of geomorphology and geology on flood hazards. Our systematic geomorphic assessment focused on quantitative morphometric techniques as this approach has been illustrated in previous studies to be sensitive to changes in the boundary conditions by distinguishing zones subject to differential rock uplift rates and subsequent complex erosional regimes^29–36^. Topographic features of the study area were extracted in order to provide an overview of topographic heterogeneities and incision patterns that change due to variations in tectonic and climatic boundary conditions. Complimentary to the geomorphic analysis, we also carried out field and sedimentological studies because the stratigraphic successions are archives of past climatic and tectonic history and preserve the records of paleo-flood events. Our study shows that in intermontane basins affected by tectonic and climatic forcing, the response of the landscape can act as a driving mechanism for flooding despite low rates of rainfall in the orogen interior.

### Drainage basin disequilibrium, geomorphic expressions and stratigraphic architecture

Landscapes are composed of streams that erode bedrock and transport sediment out of the fluvial system and hillslopes in order to attain equilibrium. Any change in the boundary conditions forces a landscape into disequilibrium that reflects on the morphology of streams that drain the region. Here we show the feedback of topography to changes in tectonic and climatic boundary conditions by analyzing channel elevations through χ that serves as a valuable metric for the state of topographic disequilibrium^37–40^.

Variations in boundary conditions can be characterized by the steepening of channel slopes that has a first-order dependence on discharge or its proxy, the drainage area^39^. Anomalies in χ arise from temporal changes in drainage area which can be caused by river capture or divide migration that reorganizes the drainage network, promoting a disequilibrium state of the river channels^40^. Theoretically, χ depends inversely on drainage area, in that a loss of drainage area would be characterized by higher χ values and conversely an increase in drainage area would lead to a decrease in χ values^39^.

**Figure 4:**
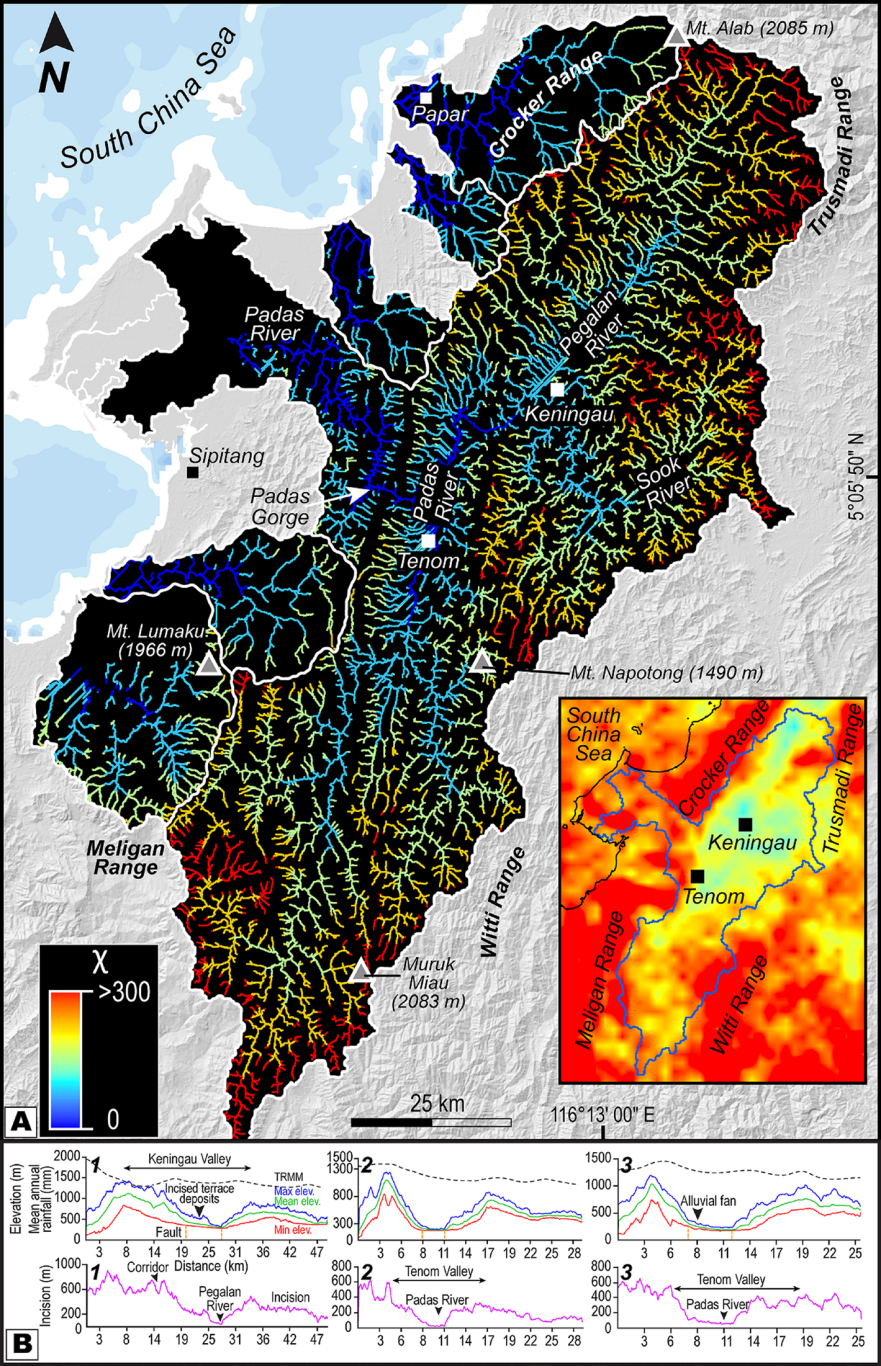
Map of χ highlighting disequilibrium in basins and topographic profiles. (**A**) The basins illustrate differences in χ across water divides (solid white line) showing disequilibrium in network geometry. The large and long basin is quickly losing drainage area to the smaller opposing basins demonstrated by high-χ upper reaches that are vulnerable to capture by the streams of the smaller basins as the divide migrates in a SE direction. Inset map shows precipitation gradient across the orographic barrier promoting drier conditions within the large and long basin. Base map is a SRTM^104^ (Shuttle Radar Topographic Mission) Digital Elevation Model (DEM) of 30 m (1-arc second) spatial resolution (SRTM 1 Arc-Second Global elevation data courtesy of the U.S. Geological Survey, https://lta.cr.usgs.gov/SRTM1Arc). Geographic Information Systems (GIS) software ESRI ArcGIS^105^ version 10.2.1 (http://www.esri.com/software/arcgis/arcgis-for-desktop) was used to create the map of χ and inset map. Figure labels were added using Adobe Illustrator^106^ version CS5.1 (http://www.adobe.com/products/illustrator.html). (**B**) Swath topographic profiles along three selected transects highlighting variations in topography and incision trends. Note the limits of the valley are confined by known faults. Refer to Fig. 2A for location of bounding rectangles used for extraction of profiles. Figure labels were added using Adobe Illustrator^106^ version CS5.1 (http://www.adobe.com/products/illustrator.html).

Spatial distribution of χ in the studied drainage basin and surrounding watersheds to the north highlight anomalous variations in χ values marking disequilibrium in the river network topology and geometry (Fig. 4A). The upper reaches of the tributaries of the Pegalan River and Padas River flowing on the northern flank of the Keningau and Tenom Valley respectively, demonstrate high values of χ as opposed to the lower values identified in the upper reaches of the tributaries of the basins on the opposing flank (Fig. 4A).

Unpaired terraces on opposite sides of stream valleys or along the insides of meander bends have been interpreted to indicate that the stream has downcut the valley over time leaving terraced deposits (cut-and-fill terraces) at different elevations. The Keningau Plain shows an asymmetrical sedimentary filling composed on the eastern side by fluvial and colluvial deposits (Fig. 3). The AMS ^14^C dating of *in-situ* wood fragments extracted from the cut-and-fill terraces (Fig. 3B) indicates an age of ca. 37,900 years BP (Table 1).

**Table 1:** ^14^C AMS dating.

Sample type	Date BP	BP error	δ^13^C (±0.1‰)	Calibrated age: 2 sigma 95.4%
Wood	37,900	900	−27.3‰	[cal BC 40841: cal BC 39548] 1, [cal BC 41498: cal BC 38586] 1,

Well-rounded pebbles and cobbles showing cross bedding and erosional surfaces between different fluvial units are observed (Figs 3A,C,D and 5). Locally, sand sheets, and fine sandy and silty lenses also occur (Fig. 3B and H). Laterally, the fluvial units are interrupted by a NE-SW oriented step fault visible on the 3D perspective view of the Keningau Plain (Fig. 3) and are in contact with Holocene fluvial deposits that occur along the major river of the valley (Figs 3C and 5). Facies analysis shows three main types of sedimentary settings: (i) braided rivers (channel fills with cross bed, lag deposits and overbank deposits from floods) (Figs 3C,D and H), (ii) alluvial fans (Fig. 3F,G and H) and (iii) debris flows (Fig. 3A and E). They are characterized by four main lithofacies (i.e., *Gms*, *Gm*, *Gt*, *Sp* following Miall^41^). Table 2 gives a summary of the lithofacies, sedimentary structures and the environment of deposition linked to the field photographs shown in Fig. 3(A–H). The lithofacies *Gms* defines massive boulders with matrix-supported gravel interpreted as debris flow deposits (Figs 3E and 5). *Gm* corresponds to massive or crudely bedded gravel that could represent lag deposits (Figs 3C and 5). *Gt* characterizes gravels with cross-stratification interpreted as minor channel fills (Fig. 5). *Sp* is characterized by medium to very coarse sand containing pebbles and might have been deposited as linguoid and/or transverse bar (Fig. 5). The sedimentary facies is interpreted as coarse fluvial deposits transported as bed-load in low sinuosity rivers (e.g. braided river systems). The morphology of this facies and contact relationship with the underlying and overlying facies types suggest that it was mobilized only during periods of flooding, and moved downstream as a sand sheet covering the channel. This facies is characterized by sharp erosional contact in the bottom surface and gradational contact with top bed. Corroboration of the sediment characteristics and bedform morphology with that of typical flood-deposits of modern river channels^42–44^ also affirm this inference.

**Figure 5:**
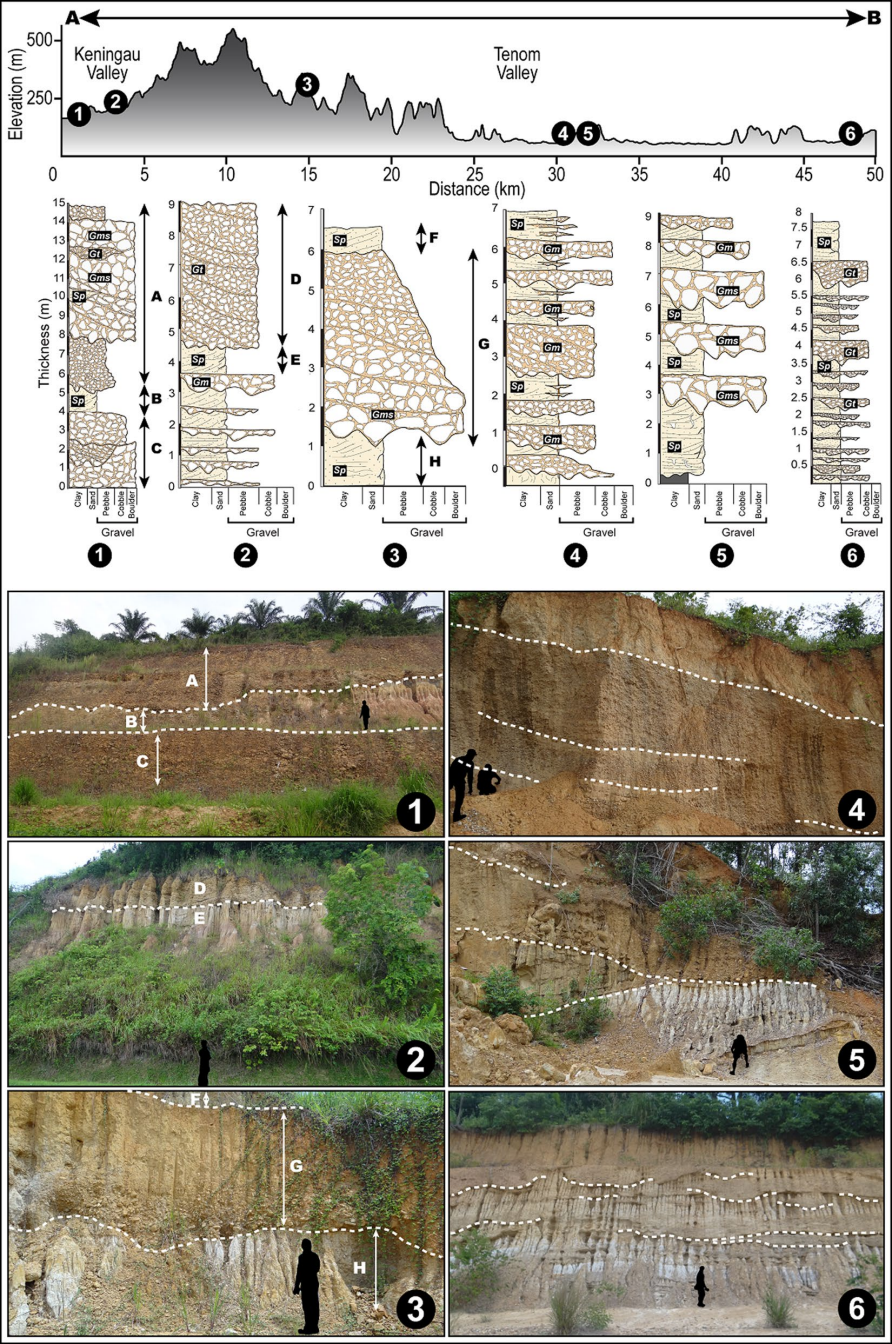
Sedimentary architecture and lithofacies. Stratigraphic logs of selected outcrops along with corresponding field photographs highlighting important variations in lithofacies. Also shown is a cross sectional topographic profile indicating altitude of logged outcrops in Keningau and Tenom Valleys. Refer to Fig. 6A for transect of topographic profile, location of outcrops and photographs. Refer to text for explanation of lithofacies codes. Sedimentary logs were drawn and figure labels were added using Adobe Illustrator^106^ version CS5.1 (http://www.adobe.com/products/illustrator.html).

**Table 2:** Lithofacies, sedimentary structures and environmental setting of selected outcrops along Keningau and Tenom Plains.

Photographs (Fig. 3)	Lithofacies	Sedimentary structures	Environment of deposition
A	Cobbles and pebbles	Cross beds and planar beds	Debris flow
B	Medium to fine sand, carbonaceous clay and organic matter (wood)	Planar beds	Small ephemeral channels
C	Matrix of medium to coarse grained sand and gravel	Trough cross beds	Channel fills and channel lag
D	Gravels and sand	None, erosional surface	Channel lag and flood plain
E	Massive boulders and matrix supported gravel	None	Debris flow
F	None	None	Alluvial fan
G	Clast-supported massive boulders	None	Alluvial fan
H	Gravels and sand sheets	Planar bed in the sand	Alluvial fan andoverbank flood

The Pegalan River takes a straight course flowing through a narrow and deep channel with an abrupt scarp on the eastern flank. The Padas River in the Tenom Plain is a meandering river, with several oxbow lakes, which displays a similar abrupt scarp on the eastern flank (Figs 2 and 3). On the western flank of the Keningau Plain, triangular facets are well expressed (Fig. 3), indicating recent rejuvenation of this zone likely related to active tectonic events accommodated along normal faults^45–49^.

In general, tectonic activity can generate alluvial fans at the foot of orogens associated with fault- and fold-bound mountain fronts^50, 51^. The Late Pleistocene cut-and-fill terraces are archives of global–scale climate change^52^. From north to south, the Tenom Plain (Fig. 3) shows a sedimentary fill composed of alluvial fans located on the west flank of the valley with multiple stages of progradation extending in NW-SE direction and Holocene fluvial deposits along the Padas River (Fig. 2B). The lengths of alluvial fans shows a decrease from south to north (Fig. 3) with the largest fans reaching 5 to 6 km in the southern compartment, and in the northern compartment, the length reaches 2 km.

### Imprints of active tectonics

Three blocks have been selected in order to analyze topographic variations. The plot for maximum elevation corresponds to the ridgelines and the curve for minimum elevation depicts the valley floors or river beds. For each swath profile, an asymmetrical valley shape can be observed (Fig. 4B). Swath profile 1 shows the ~20 km wide, 550 m deep Keningau valley, evolving after the Pegalan Gorge (Fig. 2) into an asymmetrical valley (block 2 and 3) that shows low relief on the southern flank. In blocks 1 and 2, the valley appears narrow and more asymmetrical than in block 3. An estimate for fluvial incision in a given area is provided by the arithmetic difference (residual) between the curves for maximum and minimum elevations. Incision was determined using the envelope surface (i.e., maximum elevation; the surface constructed from ridgelines) and the sub-envelope surfaces (i.e., minimum elevation; the surface constructed from channel-bottom points)^53, 54^ produced with the highest and lowest points of the topography, respectively. Incision or “geophysical relief”^55^ is defined as the residual of the envelope surface and the current topography^56^. It yields minimum values because the undissected surface remnants are not present everywhere and interfluves are arbitrarily considered to be uneroded. Since river erosion is steered by the constant interplay between uplift and climate^57^, incision can be a proxy for tectonic uplift, base-level lowering, or decreases in sediment loads. Within the basin, incision increases from the northern flank of the valley to the southern flank. For the main valley in each block, the incision reaches 550 m in the block 1; however, within blocks 2 & 3, the incision is ~300 m. The flanks of the valley are controlled by faults and the present-day rivers show a deviation toward the right flank (Fig. 2).

**Figure 6:**
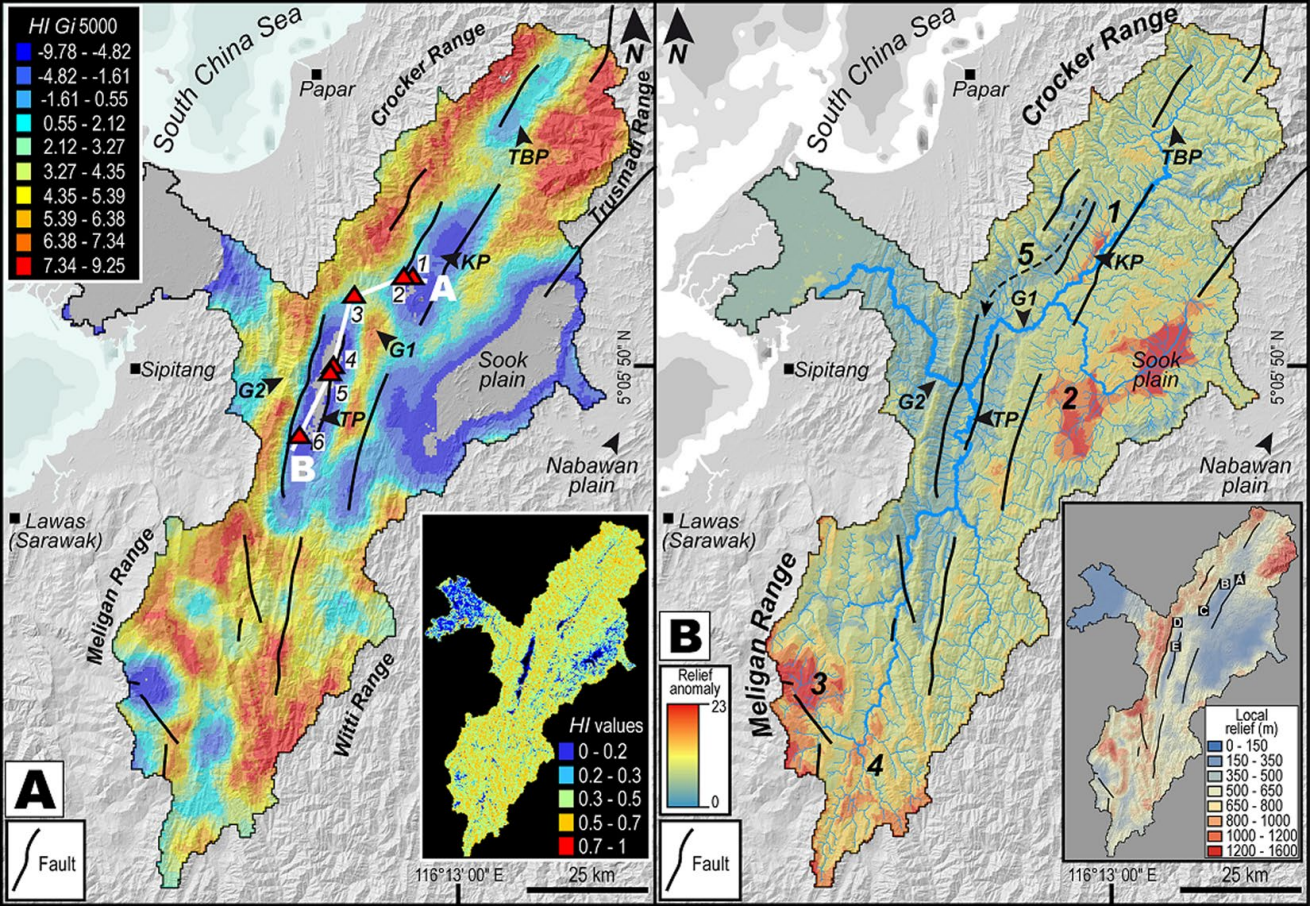
Spatial variation of topography. (**A**) Gi* statistics estimation for values of basin-wide hypsometric integrals. Hotspots are shown by warm colors and indicate high values of hypsometric integrals. Coldspots are represented by cool colors and indicate low values of hypsometric integrals. Distinct clusters of hotspots and cold spots are visible. Note fault bound cold spot located in the Meligan range forming the headwaters of the streams flowing in the Tenom Plain. Inset figure shows the spatial distribution of hypsometric integrals using an analysis grid of 1 km^2^, prior to treating it with Gi* statistics (see methods). Note that they do not show any pattern pertaining to clusters of high and low values. Base map is a SRTM^104^ (Shuttle Radar Topographic Mission) Digital Elevation Model (DEM) of 30 m (1-arc second) spatial resolution (SRTM 1 Arc-Second Global elevation data courtesy of the U.S. Geological Survey, https://lta.cr.usgs.gov/SRTM1Arc). Main and inset maps were created using Geographic Information Systems (GIS) software ESRI ArcGIS^105^ version 10.2.1 (http://www.esri.com/software/arcgis/arcgis-for-desktop). Figure labels were added using Adobe Illustrator^106^ version CS5.1 (http://www.adobe.com/products/illustrator.html). (**B**) Relief anomaly map of the catchment highlighting four major anomalous zones numbered accordingly (1–4). Note the congruity of zone 3 and 4 with the Gi* hotspot map indicative of a low relief relict surface perched at high elevations in the Meligan Range. Inset map shows local relief demonstrating higher relief in parts of the Crocker, Meligan and Trusmadi Ranges. Base map is a SRTM^104^ (Shuttle Radar Topographic Mission) Digital Elevation Model (DEM) of 30 m (1-arc second) spatial resolution (SRTM 1 Arc-Second Global elevation data courtesy of the U.S. Geological Survey, https://lta.cr.usgs.gov/SRTM1Arc). Main and inset maps were created using Geographic Information Systems (GIS) software ESRI ArcGIS^105^ version 10.2.1 (http://www.esri.com/software/arcgis/arcgis-for-desktop). Figure labels were added using Adobe Illustrator^106^ version CS5.1 (http://www.adobe.com/products/illustrator.html).

Topographic variations observed in the swath profiles were further confirmed by the spatial distribution of hypsometric integrals using kilometric-scale (1 km^2^) analysis grid and the resultant maps were subjected to spatial auto correlation (Moran’s *I*) and Getis–Ord (Gi*) statistics in order to efficiently extract abrupt topographic anomalies. Hypsometric integrals reveal complex interactions between erosion and tectonics, and can be positively correlated with uplift rates^33, 58–61^. Hypsometric integrals are thought to be affected by basin parameters such as geometry, area and rapid lowering of basin elevations^33, 62–64^. By computing the hypsometric integral of each square of the analysis grid, we obtained a regular distribution of values that is independent of drainage area and basin geometry. Because hypsometric integral computation is implemented to individual squares rather than a single value for the entire basin following the conventional method, high values can occur together with low values depending on the location of the square within a dissected zone of the landscape or in a flat zone. A spatial pattern of clusters of high and low values together can correspond to regions experiencing rapid changes in elevation and incision patterns due to tectonic and climatic variations. The distribution of hypsometric integrals in the basin does not reveal precise trend of spatially grouped clusters along adjacent cells (inset of Fig. 6A). Due to the lack of patterns or distinct clusters in hypsometric integral values, we applied spatial autocorrelation statistics to extract high and low values of hypsometric integral. The spatial autocorrelation measures the degree of similarity of spatially distributed values of a single variable within their neighborhood^33, 65–68^. The Moran’s *I* scores of the catchment (*I* = 0.489968; *E* (*I*) = −0.000022; *Z* score = 98.005668; *p* value = 0.000000) show that the values are positively correlated and therefore distributed spatially in clusters. Although used to test spatial autocorrelation, Moran’s *I* does not reveal the locations and clusters of high and low values. To determine these values around a fixed location and to map the clusters, we used Getis–Ord^69^ (Gi*) statistics. Our results show evident hotspots of high values of hypsometric integrals and cold spots of low values of hypsometric integrals (Fig. 6A). The hotspots are clustered locally in the fold and thrust belts of the Crocker, Trusmadi, Meligan and Witti Ranges, whereas cold spots are observed in the four Quaternary plains. Two clearly pronounced zones are identified with high hypsometric integral values, Gorge 1 and Gorge 2 (G1 and G2) (Fig. 6A), characterized by deep valleys, of more than 600 m depth. It is interesting to note the presence of cold spots at the headwaters of the Padas River to the south (Fig. 6A).

An analysis of the local relief (inset in Fig. 6B) was conducted in the studied catchment in order to produce an overview of the spatial variation in fluvial dissection induced by changes in boundary conditions. The results indicate that the highest values of relief occur in the Crocker, Meligan, Trusmadi and Witti Ranges, and are consistent with the known faults in the basin (inset of Fig. 6B). However, anomalously low local relief in the headwaters of the Padas River in the Meligan and Witti Ranges, in the southern segment of the basin required mapping of these anomalies through the construction of a relief anomaly map. The relief anomaly analysis has the capability to highlight elevated landscapes displaying low-amplitude relief^70, 71^. The spatial distribution of relief anomaly (Fig. 6B) highlights five major zones of anomalous values. The first one corresponds to uplifted terrace deposits in the Keningau plain (western bank of the Padas River indicated by 1 in Fig. 6B) (Figs 3 and 5). Sook plain is the second (2 in Fig. 6B), characterized by a flat zone filled by Quaternary sedimentary deposits, the third and fourth zones are perched atop the Meligan and Witti Ranges respectively (3 and 4 in Fig. 6B) and are located in the southern part of the drainage basin (Fig. 6B). The third and fourth zones correspond to the headwaters of the Padas River. Mid way of the eastern streams of Keningau Plain, a corridor indicated as zone five, showing very low relief and connected to Tenom Plain can be observed (5 in Fig. 6B).

**Figure 7:**
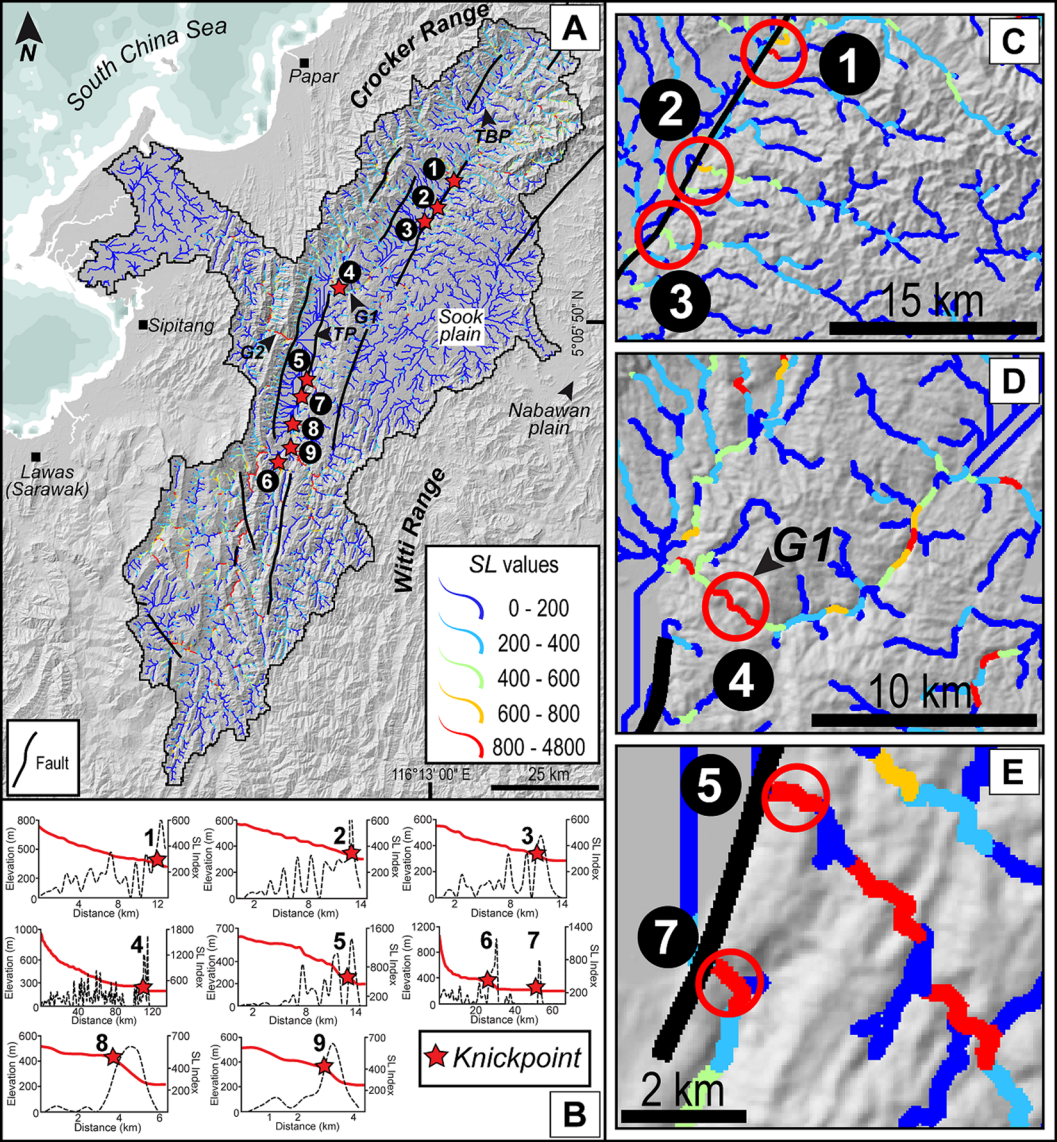
Stream profile anomalies. (**A**) Color coded *SL* index of the drainage network of the studied catchment. Also shown are faults and structural lines following Fig. 2B. (**B**) Stream longitudinal profiles and *SL* anomaly profiles of selected tributary streams of the studied drainage basin. High peaks of *SL* corresponding to knickzones are consistent with the location of known faults. (**C–E**) Close-ups showing the influence of faulting on high values of *SL* (C and E) and high *SL* values in the Gorge 1 (**D**). Base map is a SRTM^104^ (Shuttle Radar Topographic Mission) Digital Elevation Model (DEM) of 30 m (1-arc second) spatial resolution (SRTM 1 Arc-Second Global elevation data courtesy of the U.S. Geological Survey, https://lta.cr.usgs.gov/SRTM1Arc). Maps (**A**–**E**) are created using Geographic Information Systems (GIS) software ESRI ArcGIS^105^ version 10.2.1 (http://www.esri.com/software/arcgis/arcgis-for-desktop). Figure labels were added using Adobe Illustrator^106^ version CS5.1 (http://www.adobe.com/products/illustrator.html).

In landscapes displaying topographic variations through specific changes in tectonic or climatic forcing, the streams draining therein would reflect anomalous breaks in topography known as knickpoints and knickzones^36^. These anomalies indicate disequilibrium along the channel profile and mapping of these anomalies is viable through the stream length–gradient index^72^ (*SL*). Peculiarities in the stream longitudinal profiles with an array of knickzones are noticed in a basin–wide *SL* map constructed in this work (Fig. 7). Vertical displacements observed from river profiles of the Pegalan River and its tributaries reach more than 100 m with gradient index peaks exceeding 500 (Fig. 7B,C), along the Padas River and its tributaries the longitudinal profiles show at least 150 m displacement with *SL* gradient index peaks between 600 and 1700 (Fig. 7B,E). Gorge 1 and Gorge 2 are characterized by deep incision (>600 m) and show high *SL* values (>800).

**Figure 8:**
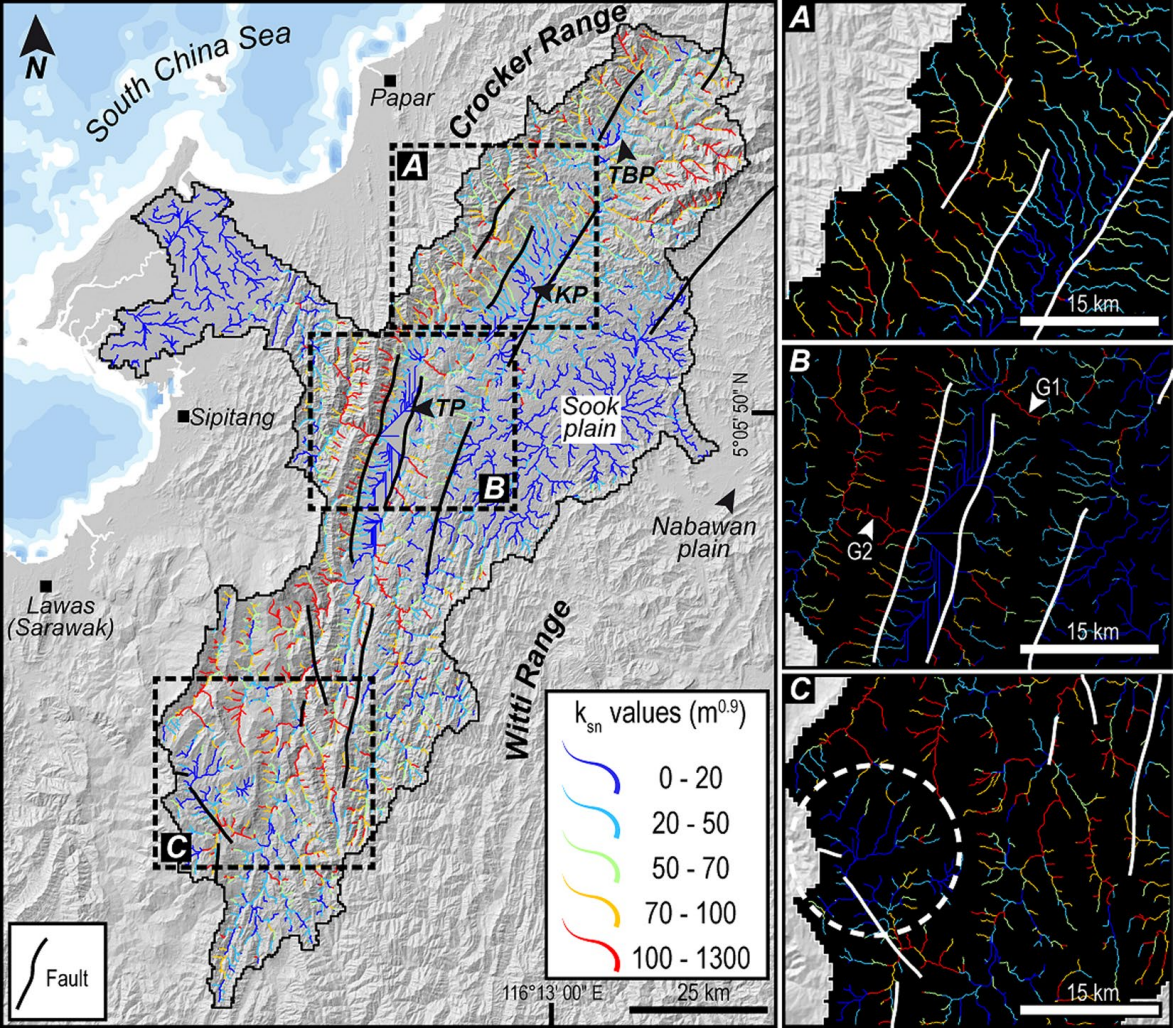
Channel network steepness. Map of color coded normalized channel steepness index (*k*_*sn*_) for the studied drainage basin. (**A–C**) Zooms of the dense stream network of the catchment shown as normalized channel steepness index. High channel steepness is noticed in parts of the Crocker, Meligan and Trusmadi Ranges. Note the relation of faults and high steepness along stream profiles. Also note a zone of low channel steepness observed in “C” indicated by white dashed-line corresponding to the location of low relief relict landscape seen in Fig. 6. Base map is a SRTM^104^ (Shuttle Radar Topographic Mission) Digital Elevation Model (DEM) of 30 m (1-arc second) spatial resolution (SRTM 1 Arc-Second Global elevation data courtesy of the U.S. Geological Survey, https://lta.cr.usgs.gov/SRTM1Arc). All maps shown in the figure are created using Geographic Information Systems (GIS) software ESRI ArcGIS^105^ version 10.2.1 (http://www.esri.com/software/arcgis/arcgis-for-desktop). Figure labels were added using Adobe Illustrator^106^ version CS5.1 (http://www.adobe.com/products/illustrator.html).

Normalized channel steepness index for streams of the entire catchment display varying *k*_*sn*_values (Fig. 8). The least values of *k*_*sn*_ ranging between 10 and 20 m^0.9^ are recorded in the Sook Plain and the mouth of the drainage basin. Streams recording higher values of *k*_*sn*_ are found in the Crocker, Trusmadi, Meligan and Witti Ranges with an average value of 400 m^0.9^. The segments of streams along known normal faults record very high values of channel steepness index in both gorges. It is also interesting to note the low *k*_*sn*_ values in the segments of the upper reaches (headwaters) of the streams of the Crocker, Meligan and Witti Ranges (Fig. 8).

## Discussion

Floods account for approximately 40% of all natural disasters occurring in both developed and developing countries^73^. The repercussions of massive floods could be catastrophic especially in highly populous developing regions such as Southeast Asia where frequent and severe natural disasters are rather common^74^. According to the United Nations Office for the Coordination of Humanitarian Affairs, an estimated ~9.6 million people are affected by flooding in Southeast Asia^73^ annually. A salient characteristic of the economies of developing countries is their over-dependence on natural resources such as land for agriculture, forestry, mining and industrialization. In Borneo, due to thick tropical rainforests, hostile terrain encompassing rugged mountains and deep valleys in the island interior, economic activities and urbanization remain confined to coastal regions and lower floodplains. Owing to increased infrastructure-development, scarcity of suitable land for industrialization, habitation and recreation in the coastal areas has compelled anthropogenic invasion into the continent interior^28^.

Our results illustrate that in the orogen interior, the geomorphology and geology of intermontane basins, such as in Sabah, dictate first order controls on flood hazards despite low rates of precipitation. Thus, assessing the adverse effects of tectonic and climatic forcing on landscape evolution and consequent geology is imperative to attenuate socio-economic impacts in the future.

Numerically modeled simulations and laboratory physical models^1–4^ have reported the response of orographic effect and progressive tectonics on drainage divides with an incomprehensive documentation of geomorphic and stratigraphic response of the leeward-side of the orogen in consequent natural landscapes. Recent progress in landscape modeling and topographic analysis techniques has led to robust identification of river catchments that experience effects of orographic precipitation and uplift and result in water divide migration. One such technique is the surrogate to steady–state channel elevation, χ, that has proved its sensitivity to detect loss of drainage area and disequilibrium in river network topology^37–40^. Our results from a rain–shadowed intermontane basin in NW Borneo show low χ values of streams in the leeward-side of the orogen as compared to the high values in the windward-side. We attribute this phenomenon to lateral migration of the drainage divide across an orographic barrier that forms a precipitation gradient (Fig. 4) and indicate vulnerability of the low χ streams to capture events and consequent loss of drainage area to opposing catchments. Across a drainage divide, in order to achieve equilibrium, the motion of the divide would be in the direction of larger χ^39^. The northern drainage divides contained in the Crocker Range and Meligan Range appear to migrate from the moisture-laden windward-side toward the drier side of the orogen, since the disequilibrium manifest in the streams provides indication on the direction of divide motion (Fig. 4A). The migration of the divide could lead to discrete capture of the streams of the Pegalan and Padas Rivers with higher χ by neighboring streams in the north with lower values of χ. Another important diagnostic feature of basins losing drainage area is the low channel steepness in the headwaters of the streams with high χ^40^. This is particularly evident in the *k*_*sn*_ map (Fig. 8). The upper reaches of the northern flank of the Pegalan River and Padas River show low values of channel steepness in the headwaters (Fig. 8). Many of the isolated long tributaries of the northern flank of the Pegalan River show very little to no side channels. The drainage network of the Pegalan River and the east end of the Padas River displays a parallel pattern perpendicular to the drainage divide. These systems of parallel streams indicate small catchments. The small size of catchments on the drier side of the orogen has been previously elucidated through laboratory models as a characteristic of divide migration^4^. This could suggest an inward migration of the lateral divides and could lead to a loss of drainage area for the side channels, thus effecting erosion capabilities and reducing erosion rates. Locally, within the studied drainage basin, possible dynamic reorganization of the river network can be observed especially in the eastern flank of the Tenom Valley. The tributaries of the Sook River appear to be losing drainage area in the upper reaches as indicated by high χ values and suggest the vulnerability to be captured by the short tributaries of the Padas River on the opposing flank that is apparently gaining drainage area. The local variations in χ values could be associated with disequilibrium induced in the system in the form of deformation along faults that generates variable topography throughout the interior of the studied drainage basin. It can be inferred that under the influence of a precipitation gradient and tectonic forcing, the basin is losing drainage area eventually leading to lower erosion rates that could exacerbate surface uplift producing higher elevations and an unstable landscape. The uplift could promote rejuvenation of older dislocations, gravity-driven normal faulting, faceted spurs, alluvial fans and debris flows as evidenced in the Keningau, Tenom and Tambunan Valleys (Fig. 3).

In Borneo, there were climatic swings during the Pleistocene with the climate alternating between short, warm, wet periods to long, cold, relatively dry periods every 100,000 years^75^. Mount Kinabalu is thought to have been glaciated during the Last Glacial Maximum (MIS2) with ice caps on the highest plateaus (>2000 m) of the range^76^. The general drop in temperature and lowering of the snow- and vegetation lines resulted in glaciation in the highest regions, frost shattering further down and debris formation in the piedmont zone^77^. Despite the drop in temperature, since Middle Miocene till present-day, Borneo is widely believed to have experienced wet climates without much interruption even during interglacial and glacial periods^78–80^. While the precipitation pattern in Borneo remained almost similar with high denudation rates since the Neogene^81^, understanding the role of tectonic uplift in providing topography to erode is critical. The large-scale uplift of the mountain ranges of entire Borneo (encompassing the ranges surrounding the studied intermontane basins) is estimated to have been at rates exceeding 7 mm a^−1^ during the latest Miocene and Early Pliocene^27, 82^. During the Pliocene, North Sabah experienced a phase of deformation triggered by trough basement uplifts and wrench faulting^83^, and terrace incision by fluvial down-cutting and deposition of coarse material in the channels was prevalent in the rivers draining the interiors of north Borneo during the Pleistocene^82^. The incision patterns in all of the swath profiles constructed in this work indicate deep incision into the alluvial fans and Pleistocene terrace deposits. This down-cutting could be in response to multi-phased tectonic events^35, 36^ and exacerbated climate forcing^84^ from Mio–Pliocene till possibly present-day. In the present–day, controls of sedimentation in the intermontane basins of Sabah is predominantly by processes that act in response to tectonically triggered and climatically exacerbated events describing a transient stage of landscape development.

Sedimentary architecture serves as an archive of past climatic and tectonic conditions^85^ and provides evidence of the after–effects of changes in boundary conditions. The stacking patterns and facies successions observed in the uplifted and exposed rocks of the catchment on the drier leeward-side of the orogen undergoing drainage loss shows massive debris flow deposits (Figs 3 and 5), which could be the result of physical disintegration following flash events. The debris flow facies constitutes mainly coarse deposits (Figs 3A,C,D,E,G,H and 5, and Table 2) that have accumulated over time and increase the porosity and permeability in the subsurface that in turn could aid in retaining flood water and recharge aquifers. Flash events in the region could be related to short periods of heavy precipitation and discharge during dry seasons or monsoons and percolation of water vertically into the subsurface can lead to a rapid rise of the local water table.

The vertical sedimentary profiles indicate lithofacies types, following Miall^41^, corresponding to a braided main river system in the Tenom plain as opposed to the meandering main river system seen in the present–day. The coupling between tectonic and climatic forcing is further evidenced from the formation of braided river systems that can support high sediment fluxes due to high stream power causing coarse valley fill during wet monsoon discharges and the presence of recurring sand sheets in the stratigraphic sections of the Tenom and Keningau Plains suggest paleo-flood events since Pleistocene times (Figs 3H and 5). As noted earlier, the migration of the main water divide could lead to an unstable landscape on the drier side of the orogen with shortening of the hillslope that facilitated mass–wasting and debris flows as seen in both Tenom and Keningau Plains. Indeed, previous studies conducted in western Kalimantan, South Borneo, witnessed Pleistocene valley fill by braided river systems that transported sediments during wet monsoons and temporarily deposited it in the river beds in the dry months^77^, further substantiating our interpretations. The presence of braided river system and thick valley infilling in NW Borneo during the Pleistocene suggest prominent feedback of the landscape to progressive forcing by tectonic and climatic processes.

Previously acquired thermochronological data from Mount Kinabalu, the highest peak in NW Borneo (Figs 2B and 3) indicate exhumation of the granite at rates of more than 7 mm a^−1^, with uplift and erosion during Late Miocene–Early Pliocene times^27^. The Mio–Pliocene exhumation was attributed to lithospheric delamination or break-off of the subducted proto-South China Sea slab which caused a large-scale rapid uplift of the entire Interior Highlands of Borneo^35, 36, 86^. Alternative postulations for the uplift in NW Borneo include subduction in the NW Borneo Trough until the Late Neogene or present-day^18, 19^, regional compression^16, 20, 21^ or extension^22^, ongoing convergence of blocks or plates, inheritance from former subduction or far-field stresses^17, 23, 24^ and possible large-scale mantle processes. Uplifting involved the Crocker, Trusmadi, Meligan and Witti Ranges that show abrupt and anomalous variations in elevation as noticed in the swath topographic profiles (Fig. 4B) and hotspot map (Fig. 6A). The hotspot map does not represent a measure of incision; hence, it does not show lithological contrasts and correlates to landscapes that are less eroded and affected by relatively high uplift that show rapid variations in elevations. The interpreted rapid uplift of NW Borneo could promote gravity-driven normal faulting (Fig. 3) owing to increased uplift as evidenced by anomalous breaks in topography forming knickzones along channels inferred from stream length–gradient index (Fig. 7), and high channel steepness along the normal faults (Fig. 8) demonstrating recent faulting and/or movement along possibly rejuvenated faults. Knickpoints and knickzones are caused by parameters such as: (a) perturbations in tectonics resulting in differential uplift, (b) bedrock erodibility variation and/or (c) drop in base-level due to high frequency sea level variations during the Quaternary^87^. The knickzones positively correlate to known normal faults and since they are at different altitudes in the Keningau and Tenom Plains, showing no correlation with any narrow elevation range, we do not favor the possibility of a drop in base-level due to sea level variations. The Quaternary plains are far from the sea and are separated by rock thresholds (Gorge 1 and 2) that further support our interpretation against a drop in base-level owing to eustatic fluctuations. The rivers containing the knickzones flow over strata containing the same rock type with no apparent lithological contrasts (Fig. 2). Therefore, the knickzones are most likely related to changes in tectonic boundary conditions. The maps of hypsometric integral hotspots, normalized channel steepness and relief anomaly (Figs 6 and 8) highlight cold spots recording low channel steepness values and low local relief at high elevations of >1000 m in the headwaters of the Padas River. This surface, indicated as zone three (Fig. 6B), could represent isolated remnants of a relict landscape that was preserved at high elevations due to rapid Mio–Pliocene uplift and erosionally unable to balance this recent uplift. Zone five, forming a corridor (5 in Fig. 6B) could have been a paleovalley that was abandoned due to a reorganization of the river network through discrete capture processes. Anomalies in relief determined through our results are expressions of recent tectonic motion with the exception of zone two that is related to the filling of the intermontane basin by alluvial sediments. The morphology of alluvial fans may be strongly controlled by tectonics^88^. In regions experiencing active thrusting and uplift, the morphology of the fan can be altered. In the Tenom Valley, the morphology of the fans in the northern segment appears short and thick and can be correlated to a differentially uplifting topography accommodated along the fault on the western flank of the valley. More spread-out, flatter, and less thick fans in the southern segment can be interpreted to relatively slower tectonic uplift and could illustrate a faster rate of sedimentation than the rate of uplift. In equatorial regions such as Borneo, geomorphic signatures of neotectonic activity can be removed rather rapidly especially when streams recording the tectonic signals flow over sedimentary rocks. Thus, we propose that recent and/or active tectonics persisted in NW Borneo and that the magnitude of uplift in this region was considerably high in order to maintain a young topography.

### Conclusions and Implications

Floods negatively impact society and often have economic and environmental repercussions. Although flooding is a natural disaster commonly presumed to be corollary to abundant rainfall, we demonstrate the role played by inherited geological and geomorphic traits of intramontane basins in retaining floodwaters despite relatively low rates of rainfall. The intramontane basin in NW Borneo, subject to tectonic activity since the Mio–Pliocene and exposed to climatic forcing at least since the Pleistocene renders a natural laboratory to test our postulation. Our results elucidate horizontal migration of the water divide, a topographic phenomenon occurring under the influence of constant rock uplift and a lateral precipitation gradient, forcing debris flows and deposition of alluvial fans on the drier leeward-side of the orogen owing to shortening that is consistent with modeled laboratory experiments. Lowered erosion rates due to loss of drainage area by shrinking of individual tributary basins facilitate a positive feedback on uplift and the resulting increase in elevation leads to gravity–driven normal faulting that produces knickzones along stream profiles, and promotes further debris flows during flash humid events. Sedimentary filling of the basin with coarse colluvium and the gradual narrowing of streams due to continuous uplift reduces the transport capacity of the streams especially in basins characterized by narrow gorges in the reaches of the main trunk stream, as shown in this study.

The coarse sedimentary facies in the subsurface accumulated over time through successive events of debris flow is characterized by high porosity and permeability forming frequently recharged aquifers. Following a wet monsoon and/or a short intense rainfall event, water can percolate vertically into the soil, leading to a rapid rise in the local water table. The major consequence of a quick increase in the upper level of the water table is that it reaches the base-level of all tributaries and main rivers, leading to rapid flooding of the inundation plain as witnessed often in the catchment of NW Borneo^28^. The framework of such basins could provide a convenient and appropriate configuration for abundant groundwater reservoirs. However, as demonstrated in this study, understanding the geological and geomorphic feedback of the landscape in order to assess flood risks prior to urbanization of areas receiving relatively lower rainfall is of tantamount significance.

## Methods

### χ Map

Steady-state channel elevation is transformed to the surrogate, χ, to determine disequilibrium in the landscape and the local drainage network, and to discern the stability of water divides. In order to construct the χ map we followed the methodology defined by Mudd *et al*.^89^. Stream network parameters such as flow direction, flow accumulation, base-level and flow paths were extracted from a SRTM (Shuttle Radar Topography Mission) digital elevation model (DEM) of 1 arc–second resolution. The DEM was pit/depression filled in order to remove voids and avoid errors while calculating flow direction and paths. χ is calculated for each pixel using the equation^89^

1 $$\chi ={\int }_{{x}_{b}}^{x}{(\frac{{A}_{0}}{A(x)})}^{m/n}dx$$

where; *A*_*0*_ is an arbitrary scaling area, *m* and *n* are empirically derived, non-integer constants and the integration is performed upstream from a base-level at *x*_*b*_ to a location *x* along the channel profile. A critical area of 10^6^ m^2^ was chosen in order to define the threshold for the minimum contributing upstream drainage area. Calculations were done assuming concavity *m/n* = 0.45 and an arbitrary scaling area (*A*_*0*_) of 1 m^2^.

### AMS ^14^C dating

The analysis of Accelerator Mass Spectrometry (AMS) radiocarbon dating was performed on sediments using fragments of wood obtained *in-situ* from the cut-and-fill terraces. Measurements were performed at the Beta Analytic laboratory of the University of Arizona, USA. Absolute dates have been calibrated using the software Calib Rev 6.0.1. The terrestrial radiocarbon calibration curve “IntCal09” for plant material and organic sediment was utilized for calibration of the ages. For the dating, a value of δ^13^C was obtained and used as proxy to indicate the origin of the organic matter^90–92^.

### Swath topographic profiles

Elevation data of complex topographies can be condensed into a single profile^93, 94^. A rectangular swath of 10 km width was chosen to extract a series of parallel profiles that are separated by 1-cell (30 m). The width of the swath used in this work was chosen as it is large enough to appropriately condense both elevated surfaces and streams, and on the other hand, this was small enough to avoid averaging topographic structures that are too oblique to the axis of the rectangular swath. Statistical parameters such as maximum, minimum and mean elevations were calculated along each swath profile within a GIS environment. The plot for maximum elevation corresponds to the ridgelines, and the curve for the minimum elevation depicts the valley floors or river beds. A measure for incision can be produced by the arithmetic difference between the maximum and minimum elevations within the longitudinal distance of the swath rectangle^71^. Annual averaged precipitation derived from the Tropical Rainfall Measuring Mission (TRMM), that was measured from 1998–2009 ^95^, was plotted along with the elevation curves.

### Hypsometric integrals

Hypsometric integrals are estimated by means of the following equation^96^

2 $$HI=\frac{Meanelevation-Minimumelevation}{Maximumelevation-Minimumelevation}$$

We computed hypsometric integrals utilizing analysis grid composed of regular squares of area 1 km^2^ instead of calculating a single value for the entire catchment. Using the kilometer-scale, significant topographic information of the area under investigation can be obtained^97^. By calculating hypsometric integral of each square, a regular distribution of values that is independent of drainage area and basin geometry is produced.

### Spatial autocorrelation (Moran’s *I*) and Getis–Ord (Gi*)

Moran’s *I* ^65^,regarded as the best-known measure to test for spatial autocorrelation^33^, is used in this study. The expected Moran’s *I* (=*E*(*I*)) is calculated assuming a random distribution of the values and is generally very close to 0. The actual Moran’s *I* (=*I*) values range between −1 and 1, whereby, if *I* is greater than 0, it shows a positive spatial autocorrelation in which similar values of high or low are spatially clustered. On the other hand, if the values are less than 0 or close to −1, it indicates a negative spatial autocorrelation showing complete dispersion of values. *Z* score and *p* value are calculated to obtain a confidence level that any pattern of positive of negative association is not just coincidence^98^. Positive *Z* scores indicate clustering, while a negative *Z* score indicates dispersion.

To determine the concentration of high or low values around a fixed location and to map the corresponding clusters, we used Gi* statistics. The Gi* statistics provides specific measures of spatial association by defining a set of neighbors for each location as being those observations that fall within a specified distance (*d*)^65^. The statistics is defined by Ord and Getis^69^ as

3 $$\,G{i}^{\ast }=\frac{{\sum }_{j}{w}_{ij}(d){x}_{j}}{{\sum }_{j}{x}_{j}}$$

where; *w*_*ij*_(*d*) are the elements of the contiguity matrix for distance *d* and *x*_*j*_ is the measured attribute of interest at location *j*.

### Stream length – gradient index (*SL*)

*SL* index was defined by Hack^72^ as;

4 $$SL=(\frac{{\rm{\Delta }}h}{{\rm{\Delta }}l})L$$

where; Δ*h/*Δ*l* is the local channel gradient and Δ*h* represents the variation of altitude for a channel of the reach with respect to Δ*l* that signifies the length of the reach. *L* is the total horizontal channel length from the divide to the midpoint of the channel reach upstream for which the index is being calculated.

### Relief analysis

We investigated the topographic features of the NW Borneo catchment (Fig. 3) in terms of relief values, focusing on the spatial variation in maximum, mean and minimum elevations. These were extracted to produce a local relief map that provided an overview of the topographic heterogeneity and river incision patterns. We computed the local relief map as the residual relief between maximum topography, which is the peak elevation displaying surfaces without fluvial dissection, and the minimum topography corresponding to the general pattern of valley bottom elevations. We constructed a relief anomaly map to highlight surfaces displaying low local relief at high elevations. It represents elevations normalized with respect to the local relief. Relief anomaly is defined by Scotti *et al*.^70^ as;

5 $${A}_{r}=\frac{{H}_{mean}}{{R}_{l}}$$

where; *H*_*mean*_ is the mean elevation of the raw topography and *R*_*l*_ is the local relief.

### Normalized channel steepness index

The slope–area regression has the following form^99^

6 $$S={k}_{s}{A}^{-\theta }$$

where *S* is the local channel slope, *k*_*s*_ is the steepness index, *A* is the upstream drainage area and *θ* is the channel concavity index. *k*_sn_ is estimated by normalizing the catchment area of a given reach and using a reference concavity (*θ*_ref_), which corresponds to the regional concavity observed in reaches unperturbed by tectonic signals. The above equation can be re-written as

7 $$S={k}_{{\rm{sn}}}{A}^{-(\theta {\rm{ref}})}$$

*θ*_ref_ of 0.45 was used for all channels to facilitate the comparison of our results. The value was chosen as it is within the range commonly perceived in bedrock rivers regardless of rock uplift rates and erosion^100, 101^.
